# A wearable IMU-based method for measuring biomechanical parameters in hammer throwing

**DOI:** 10.3389/fbioe.2026.1800086

**Published:** 2026-07-21

**Authors:** José Sánchez-Moreno, David Moreno-Salinas, Carlos Revuelta-Parra, Juan Carlos Álvarez-Ortiz

**Affiliations:** 1 Department of Computer Sciences and Automatic Control, Universidad Nacional de Educación a Distancia (UNED), Madrid, Spain; 2 Department of Sports Sciences, Faculty of Medicine, Health and Sports, Universidad Europea de Madrid (UEM), Madrid, Spain; 3 Federación Española de Atletismo (RFEA), Pozuelo de Alarcón, Spain

**Keywords:** azimuthal angles, hammer throw, inertial measurement unit, motion capture, performance, wearable sensors, throwing technique, sport biomechanics

## Abstract

**Introduction:**

The development of a low-cost system for analysis of the hammer throw technique is presented. The system is based on analyzing the information provided by off-the-shelf inertial measurement units (IMUs).

**Methods:**

Five Spanish national-level hammer throwers (2 women, 3 men) performed 6 trials with homologated implements (4 kg for women, 7.26 kg for men) at the High-Performance Sports Centre (CAR) in Madrid during regular training sessions. The IMUs were positioned at three points on the thrower’s body: right instep, lumbar zone, and wrist. All IMU raw data (angular velocities, linear accelerations, and Euler angles) were processed and visualized using Matlab software to analyze and derive additional parameters.

**Results:**

After analyzing the patterns of the raw data provided by the IMUs with professional coaches, algorithms for determining the duration of the single and double support phases for each turn were developed and described in the paper. These raw data also allow us to derive additional biomechanical parameters that help characterize the quality of the athlete’s throwing technique (wrist angular velocity and linear acceleration, pelvic tilt, horizontal azimuthal angles, arm elevation angles, and vertical foot acceleration). Validation against a VICON motion capture system showed excellent agreement for phase durations (ICC = 0.977) and angular displacements (ICC = 0.976).

**Discussion:**

The data obtained using IMUs represent only a subset of the information that can be obtained from a hammer throw using current 3D photogrammetry techniques. However, as the hammer throw is a rotatory movement, a valuable information according to the literature can be obtained from IMUs at very low cost and without access to specialized high-performance centers or biomechanics laboratories. These findings demonstrate that an IMU-based framework constitutes a reliable low-cost solution for coaches and athletes who can benefit from this technology in their regular training sessions.

## 1 Introduction

The application of hardware and software technologies to improve the performance of the athletes is an endless race, especially in the elite categories where 1 millisecond or centimeter can mean a new world record or a gold medal. Until now, the biomechanical analysis of athletes is mainly trusted to 3D photogrammetry techniques where the video recordings obtained in a training session or a competition are analyzed by sport engineers or software tools based on AI algorithms to determine the 2D/3D coordinates of the body landmarks. From these coordinates is already possible to derive biomechanical parameters (orientations of body segments, linear and angular velocities and accelerations, centers of mass, angular momentum, etc.) that are relevant to sports researchers or coaches to improve the performance of their trainees. There are other methods of extracting data by strain gauges or accelerometers fixed to the athlete body (Baca and Kornfeind, 2006; Taborri et al., 2020) or to the equipment (Umek et al., 2017). Examples applied to a throw implement, a hammer, are the works described in (Murofushi et al., 2005; Brice et al., 2008) where a strain gauge was mounted on the wire of the hammer to measure the force acting on the hammer’s head; or (Wang et al., 2016) where a load cell is built into the handle for recording the wire tension during a hammer throw. Other examples are described in (Liu et al., 2011; Zhao et al., 2011) where the strain gauges, the electronics, and the battery are introduced inside a discus to obtain acceleration and velocity data of each throw in real-time. Similar developments for shot-put can be consulted in (Song et al., 2006; Gao et al., 2009), where the shot is entirely designed and fabricated to introduce the force sensors and the associated electronic inside the implement.

Thanks to the improvements in reduction of price, energy consumption, weight and size, a technology commonly used in both civilian and military industries from years ago is now accessible to everyone for many purposes. This technology is a sensor, known as inertial measurement unit (IMU), that measures linear accelerations and angular velocities across three perpendicular axes, providing data about an object’s movement and its orientation in 3D using a combination of accelerometer, gyroscope, and often magnetometer (Kok et al., 2017). According to the information that an IMU can provide, one of its direct application fields in sports is in the four throwing events (hammer, discus, shot put, and javelin). The use of IMUs in both professional and amateur sports has been extensively justified in the scientific literature (Cuesta-Vargas et al., 2010; Camomilla et al., 2018; Alanen et al., 2021; Hindle et al., 2021; Espinosa et al., 2025). However, its application in the throwing events has not been so pervasive and direct, maybe due to the minority practice of these disciplines even within athletics.

To the authors’ knowledge, the first use of IMUs for analyzing throwing movements was reported by Särkkä et al. (2016), who embedded a sensor in a javelin tip to estimate its attitude, position, and velocity. Subsequent javelin studies include Looijen (2020), Braic (2025), and TDK (2025). For discus throwing, IMUs have been used to measure 3D kinematics (Navarro-Iribarne et al., 2022), shoulder-pelvis separation angles (Brice et al., 2018a), and support phases (Sánchez-Moreno et al., 2025). In shot-put, Saračević et al. (2018) applied an inertial suit to measure kinematic variables, and Kato et al. (2024) demonstrated that angular velocity waveforms can effectively detect the release instant.

Focusing the reader attention on the use of IMU in hammer throw, the first reference found in literature is (Wang et al., 2018), where authors propose to use an IMU to collect the vertical displacement of the thrower during the training sessions. This work is extended and improved in (Wang et al., 2023) by training deep learning models with data recollected from different sources: two IMUs (located in wrist and waist), a load cell built in the hammer handle, and a motion capture system. In (Tiedemann et al., 2022a) is described the integration of an IMU inside a 4 kg hammer head to measure the radial acceleration of the hammer in real-time; the only problem reported by the authors is that the measuring range of the accelerometer, 32 g, is almost exhausted during the experiments. A work that describes a relevant and intensive use of IMUs to obtain performance-determining biomechanical parameters in hammer throw can be found in (Tiedemann et al., 2022b). In this article is afforded the analysis of the hammer throwing movement by a full-body sensor suit that let obtaining kinetic and kinematic parameters of 23 body segments. Working with these data, the authors derive much of the literature-based biomechanical parameters of the hammer throw technique, as, for example, the double- and single-support phases durations.

However, even though the results obtained using full-body sensor suits are very exhaustive and accurate, the existing off-the-shelf inertial motion capture systems, such as Xsens® Awinda^1^, Perception Neuron^2^, or Synertial^3^ systems, require at least seventeen IMU units and complex proprietary software to construct a complete human motion model for comprehensive biomechanical analysis. These systems are not practical for a coach and an athlete in a regular training session or in an official competition for different reasons: (1) excessive setup time, 15–30 min, unsuited with the training dynamics where athletes need to maintain a warm-up state and training rhythm, making such preparation times unrealistic for routine use in athletics coaching; (2) the hardware and software complexity makes it difficult for coaches and athletes to operate these systems independently and interpret data output; and (3) the cost ranges from 25.000 to 30.000 €, which restricts these systems to specific high-performance centers or advanced biomechanics laboratories, making the access to these tech resources impossible for many coaches and athletes, typically working at athletics tracks at universities or in cities. While extensive 3D motion analyses technologies do exist, practitioners find that they are too cumbersome to be useful for training.

In this paper, a solution is presented to meet some of the practical demands in hammer-throw analysis at a cost that can be afforded by coaches and their athletes. The idea is to use a set of three off-the-shelf IMUs located on specific parts of the athlete’s body (wrist, lumbar zone, and feet) and to describe the necessary algorithms to process the raw IMU data and derive relevant kinematic parameters such as the phases of the hammer throw and the azimuthal angles. A similar idea was presented in Tiedemann et al. (2022b), but instead of a 17-IMU full-body Lycra suit, in the present work the thrower only wears a maximum of three IMUs and the initial calibration process is minimal, consisting of synchronizing the inner clock of the IMUs while the athlete stands for a few seconds in a steady-state position. Also, instructions for processing the raw data are included throughout the paper.

## 2 A brief on hammer throw technique

Hammer throw is a highly technical and complex athletics event based on throwing a heavy implement as far as possible from a circle with a diameter of 2.135 m. The hammer weighs a minimum of 7.26 kg for men and 4 kg for women. During the throwing, throwers perform a specific sequence of footwork, alternating phases with double support (DS) and other with simple support (SS), i.e., both feet in contact with the ground or only one, respectively, and performing a combined movement of rotation and translation. In fact, for a right-handed thrower, the left foot is always in contact with the circle of throw across which it translates with a series of shifts from heel to toe. During the SS phase, the hammer head reaches its highest point and during the DS phase its lowest point. This movement (turn) is repeated three or four (five is very unusual) times during the throw until the moment of the hammer release. Prior the turns, athletes perform preliminary winds (typically two or three), in which the hammer is rotated around the athlete´s head. Thus, chronologically three main parts can be identified: (i) the winds (the hammer turns around the thrower) creating conditions for entering the first turn; (ii) the turns (thrower and hammer, both turn around a common vertical axis located between the hands and the torso of the thrower); and (iii) the delivery or release (short time from the last turn until the hammer’s head is in an ideal angle and the grip is quickly released).

Based on the right foot’s takeoff and touchdown, each turn is divided into a SS and a DS phase (some authors refer to the last DS phase as the delivery phase). Considering that the thrower makes four turns, each of these turns includes four critical instants or events: 1) right foot takeoff (R_*off*_); 2) hammer head’s highest point (HP); 3) right foot touchdown (R_*down*_); and 4) hammer head’s lowest point (LP). The HP and LP are defined as the highest and lowest elevation angles of the hammer head in each turn, respectively. There is an additional critical event corresponding to the point of release (RP) and determines the end of the movement (*t*_*release*_). Thus, in a four-turn throw there are three sets <R_*off_i*_, HP_*i*_, R_*down_i*_, LP_*i*_> with *i* = 1,2,3 plus a fourth set <*R*_*off_4*_, HP_4_, *R*_*down_4*_, LP_4_, RP>.

**FIGURE 1:**
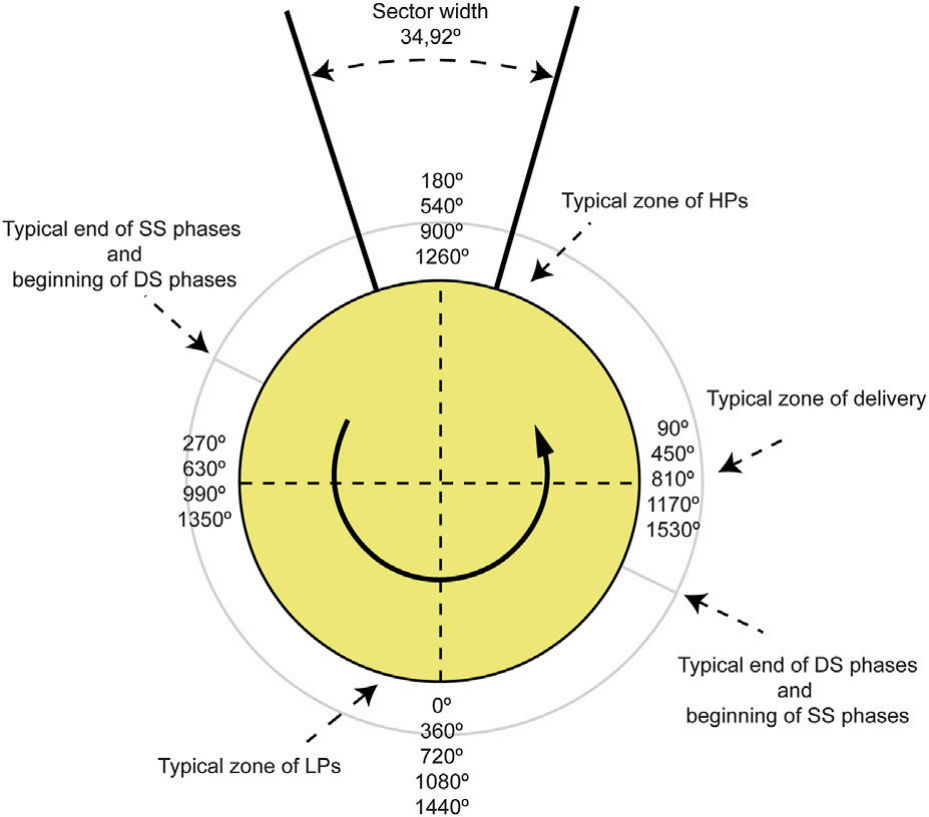
The hammer circle showing the zones of the SS and DS phases, the HPs and LPs of the hammer head, and the azimuthal angle reference system.

As the aim of the discipline is to maximize the throwing distance (women’s WR: 82.98 m, Anita Włodarczyk, 2016; men’s WR: 86.74 m, Yuri Sedykh, 1986), the thrower must try to increase the velocity of the implement during both the winds and turns. Traditionally, it has been assumed that the DS phase is more important for accelerating the ball (Pavlović, 2020), as it is the moment when the thrower can apply force with both feet on the ground. However, it has also been demonstrated that acceleration is possible during the SS phase (when neglecting air resistance and gravity effects) (Dapena, 1984). Therefore, knowledge of the parameters associated with the turns (turn time, DS, SS, LP, HP, RP, elevation angles) is fundamental for coaches to understand the behavior of the different phases. In addition, there are other parameters that can help coaches, such as the azimuthal angles. The azimuthal angle refers to the angular displacement of the ball in an overhead view during each turn. An earlier touchdown with the right foot improves the azimuthal angle and allows the thrower to accelerate the hammer for a longer time with both feet in support. See Figure 1 for a view of the hammer circle with the most common areas of the SS and DS phases, the zones of the HPs and LPs, and the azimuthal angles. A detailed review of this discipline and its ballistic, environmental, and biomechanical aspects can be found in Castaldi et al. (2022).

## 3 Materials and methods

The study developed can be divided into two phases: measurement and validation. The measurement phase involved recording data from five athletes using three body-mounted IMUs to obtain raw data for further analysis and parameter extraction.

The validation phase consisted of two subphases. An outdoor subphase for recording data from one athlete with an additional IMU placed on the hammer wire, near the ball, to verify that the wrist elevation angles could serve as a reliable proxy for the hammer head elevation angles. And a second indoor subphase in a biomechanical lab to certify that the results obtained from the IMU data in the measurement phase were valid.

### 3.1 Participants

The participants in the study were five high-level Spanish hammer throwers (two women and three men). They performed six trials each with homologated implements (4 kg for women, 7.26 kg for men) at the outdoor track and field facility of the High-Performance Sports Centre (CAR) belonging to the Spanish Sports Council (CSD) in Madrid, Spain. Except for one left-handed female athlete (Athlete ID 5), all athletes rotated to the left (left foot keeping contact with the circle). All of them used a throwing technique consisting of two preliminary swings and four turns. Table 1 presents the anthropometric characteristics of the athletes and the number of trials evaluated in the measurement phase. The performance levels of the athletes place them in the national-level category, providing a representative sample to assess whether the proposed IMU-based methodology can capture biomechanical parameters within physiologically and technically plausible ranges across different skill levels and both sexes. The throws were recorded during two training sessions in the 2024–2025 fall-winter training period, and the throwing distance was measured for all throws.

**TABLE 1:** Participating Spanish athletes’ characteristics.

Athlete	Age (year)	Gender	Body height (m)	Body mass (kg)	Long distance trial (m)	Short distance trial (m)	Mean throwing distance (m)	Trials	Official PB (m)
1	34	m	1.92	110	55.79	53.02	54.86 ± 1.028	6	63.95
2	20	m	1.82	89	51.07	46.60	49.24 ± 2.017	6	53.86
3	23	m	1.88	96	57.68	55.42	56.36 ± 0.796	6	66.27
4	22	f	1.63	76	50.13	40.31	45.66 ± 3.821	6	49.94
5	19	f	1.81	85	46.39	44.60	45.33 ± 0.857	6	46.13

The throws of the indoor validation subphase were recorded in a session at the Biomechanics Laboratory of the Faculty of Sport Sciences at the Universidad Europea of Madrid using a rubber-headed hammer of 5 kg. Athlete ID 1 was selected for the validation phase, and the number of trials was three. In this session, the distance was not registered due to the impossibility of releasing the hammer.

### 3.2 Software and hardware

The Movella DOT was selected as the inertial measurement system for the experiments (Movella, 2021). This product is a 9-axis IMU (accelerometer, gyroscope, and magnetometer), called DOT for the sake of simplicity, specifically designed to capture kinematic data from the human body at frequencies up to 120 Hz. The measurement ranges of the accelerometer, gyroscope, and magnetometer are ±16 g, ±2000°/s, and ±8 Gauss, respectively. Several factors motivated this selection: its time synchronization, setup, recording control, and BLE^4^ data export features are highly intuitive, making it accessible to non-technical users. This user-friendliness is particularly valuable for coaches and athletes, the intended end users of the system.

**FIGURE 2:**
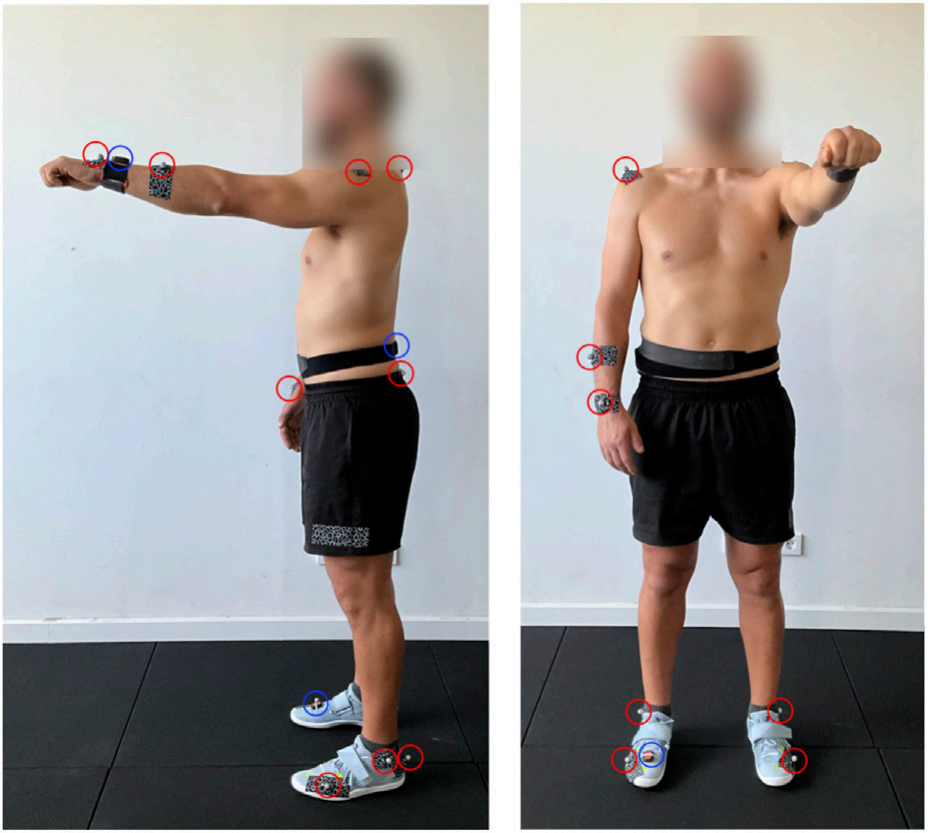
Locations of the IMUs (blue circles) and reflective markers (red circles) on the athlete’s body.

The setup employed with all the athletes consisted of three IMUs attached to the right wrist (X-axis pointing toward the shoulder and Y-axis toward the back), to the right instep (X-axis pointing toward the leg and Z-axis pointing outward from the foot), and to the lumbar region (X-axis pointing toward the head and Y-axis toward the left arm). For the left-handed athlete (Athlete ID 5), who rotates to the right with the right foot keeping contact with the circle, the IMUs were placed on the left instep and left wrist, mirroring the setup used for right-handed throwers. The data were processed as if the athlete were right-handed to enable direct comparison across all participants. Figure 2 shows the location of the IMUs on Athlete ID 1. The selection of these three locations was driven by the biomechanical information each placement provides: the wrist IMU captures elevation and azimuthal angles that serve as a proxy for the hammer head kinematics (see Section 5.2), as well as the angular acceleration peak used to detect the release instant; the right instep IMU provides the vertical acceleration needed to identify the takeoff and touchdown events defining the SS and DS phases; and the lumbar IMU measures pelvic inclination during the turns. This minimal configuration was designed to capture the most coaching-relevant parameters while maintaining a practical setup for regular training sessions, as discussed in Section 1.

The IMUs were set up to sample at 120 Hz, and the raw data recorded were Euler orientation angles in the ENU convention (in degrees), along with linear accelerations (in m/s^2^) and angular velocities (in °/s), both expressed in the IMU reference frame. The IMUs incorporate internal Kalman filtering to compute a statistically optimal 3D orientation estimate with high accuracy and no drift for both static and dynamic movements. However, because the hammer cage surrounding the athlete contains ferromagnetic materials, the magnetic field can become distorted, causing errors in the measured orientation. To correct these magnetic disturbances, a calibration of the IMU magnetometer must be performed when the training location changes (for example, when moving to another track and field facility). The magnetometer calibration, synchronization, and recording control were performed using the manufacturer’s app installed on a tablet. The software used for processing the raw data from the IMUs was MATLAB 2024b.

The indoor validation facilities consist of a VICON^5^ motion capture system equipped with seven Vantage series cameras (V5 and V8 models). Data processing and analysis were performed using two primary software platforms: Vicon Nexus, which serves as the core framework for data acquisition, and Vicon ProCalc, which provides the necessary tools for creating custom variables and adapting the biomechanical model to the specific requirements of the study. Figure 2 shows the reflective markers on Athlete ID 1.

### 3.3 Methodology

Before each session, the internal clocks of the three IMUs were synchronized. Additionally, before each recording, the athlete stood in a static position at the initial throwing stance, holding the arm horizontally (see Figure 2) and aligned with the 0°–180° axis of the circle for several seconds to allow for IMU offset calculation and baseline calibration.

After each session, the raw data corresponding to each throw were downloaded from the IMUs in CSV format (three IMUs, so three files for each throw) and imported into Matlab for their processing. Each row of the CSV files was composed of timestamp, Euler angles, linear accelerations, and angular velocities (a total of ten columns). No additional filtering was applied to the exported data. The Movella DOT firmware processes all sensor signals through an internal pipeline (Movella, 2021; 2023): accelerometer and gyroscope data are sampled and calibrated at 800 Hz, then low-pass filtered with a cutoff frequency chosen to preserve signal fidelity for movement analysis applications. The filtered data are processed by a Strapdown Integration (SDI) algorithm that reduces the output rate while preserving accuracy through coning and sculling compensation, avoiding the aliasing effects associated with conventional linear down-sampling. Finally, a Kalman filter (Movella, 2021; 2023) computes drift-free 3D orientation estimates from the fused inertial and magnetometer data. The sensor does not provide access to unfiltered raw data. The Movella DOT offers two filter profiles: General, which assumes moderate dynamics and a homogeneous magnetic field, and Dynamic, designed for fast and jerk-like motions of short duration. The Dynamic profile was selected because its assumptions match the characteristics of hammer throwing. The sensor bandwidth (gyroscope: 255 Hz at −3 dB; accelerometer: 324 Hz at −3 dB) is well above the frequency content of the biomechanical parameters derived in this study.

As mentioned before, synchronization of the IMUs’ internal clocks was performed before starting each session using the manufacturer’s app. However, it was noticed that due to BLE latency, the IMUs might not start recording at the same time, with offsets ranging from 1 to 14 sampling periods (8.3–116.7 ms, *h* = 0.00833 s) between the wrist IMU and the other two sensors. This issue was addressed in Matlab by a pre-processing procedure consisting of a fine synchronization of the three IMUs using the wrist sensor as the temporal reference. First, the procedure calculated the initial time offset in samples between each IMU and the wrist IMU by comparing their timestamps. To align the data streams, the procedure identified the IMU with the largest delay and trimmed the initial samples from all IMUs accordingly, ensuring they all started at the same time instant. Finally, the offsets were recalculated to verify synchronization. In all trials, the residual time offset between all IMU pairs was zero samples, confirming exact temporal alignment of the three data streams at the sampling resolution of 8.33 ms (120 Hz). Once the fine synchronization of the IMUs was done, data were ready for final processing and generation of results.

## 4 Processing and generation of results

To obtain useful and relevant parameters from the athlete movement, the first step is to detect the start and the end of the signals with useful information of the movement. Once this temporal window with valid information is delimited, the analysis of the signals is afforded.

### 4.1 Delimitation of the movement

**FIGURE 3:**
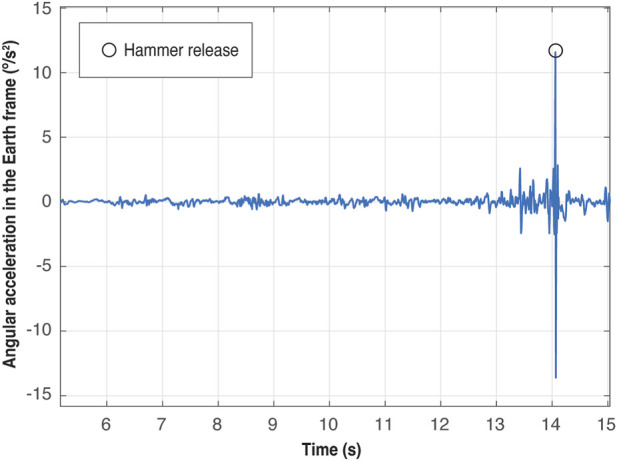
Detection of the hammer release using the angular acceleration of the IMU located at the wrist.

The end of the throw is always defined by the time instant, *t*_*release*_, at the point of release. As well as in (Tiedemann et al., 2022b), the maximum peak of the angular acceleration of the wrist in the Earth reference system was used to characterize this event. As the IMU provides the angular velocities, it is necessary to differentiate it to get the acceleration. An example of the detection of the hammer release in a throw of the Athlete ID 1 is presented in Figure 3.

Once *t*_*release*_ is known, the start of the movement, *t*_*start*_, is obtained. The common consensus to define the start is to consider it as the first time the right foot takes off (the start of the first SS phase); however, this criterion eliminates the initial DS phase that occurs before the SS of the turn 1. To avoid the elimination of this initial DS phase, we have considered that the temporal window of the movement must start at the time instant in which the elevation angle of the wrist IMU, that is, the Euler-Y angle, crosses 0° before the first takeoff, that is, *R*_*off*_1_.

**FIGURE 4:**
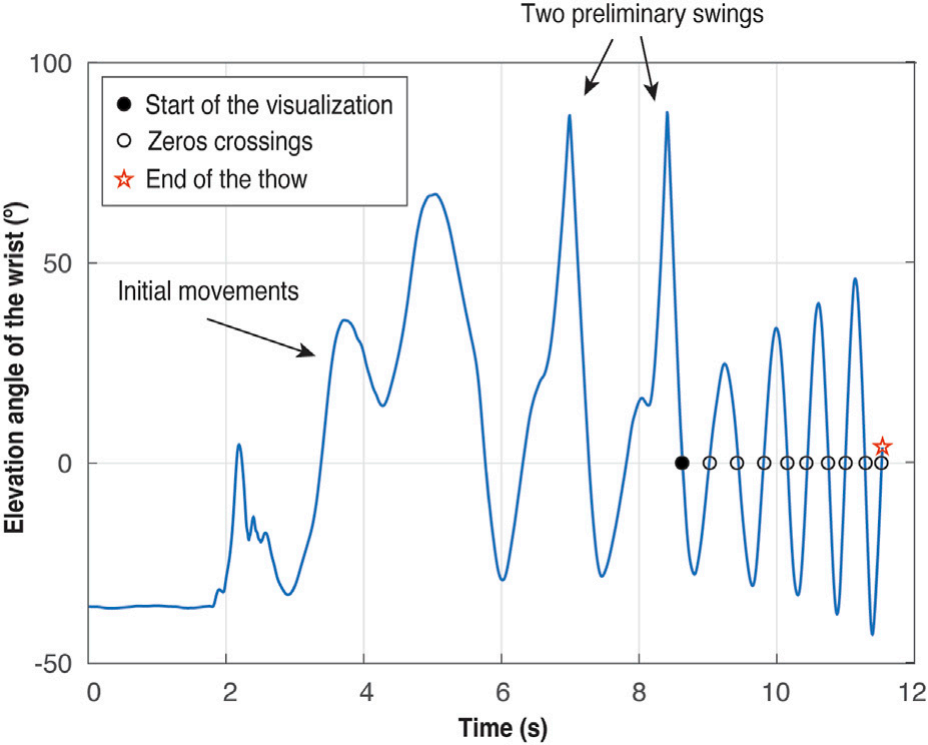
Detection of the start time for signal visualization by analyzing zero crossings of the wrist elevation angle.

The point *t*_*start*_ is determined by analyzing backwards the sign changes in the wrist elevation angle from *t*_*release*_. First, the algorithm identifies the zero-crossing points from the beginning of the signal until the release instant. To isolate the four complete turns characteristic of hammer throwing, the last 9 or 10 zero-crossings were selected depending on the change of the sign of the elevation angle at release (10 crossings if positive, 9 if negative). The first zero-crossing point of this selection is designated as the start of the visualization, ensuring that the analysis captures the complete rotational phase while excluding preliminary movements. For throwers performing three turns, it is necessary to select the last 7 or 8 zero-crossings instead. Figure 4 presents an example of the detection of *t*_*start*_ for a movement of athlete ID 1 composed of two swings and four turns. The initial movements with the hammer, the preliminary swings, and the turns can be appreciated. In this example, 10 zero-crossings are counted because the elevation angle is positive at release.

It must be noted that the true duration of the throw is not defined from *t*_*start*_. According to the literature, the duration is defined as the elapsed time from the first takeoff (R_*off_1*_), that is, *t*_*Roff_1*_, to *t*_*release*_. Thus, *t*_*duration*_ *= t*_*release*_ *-t*_*Roff_1*_.

### 4.2 Detection of SS and DS phases

Once the temporal window has been defined, it is necessary to detect the phases of the throw. As mentioned before, four throws are composed of four consecutive pairs of SS-DS phases: SS1-DS1, SS2-DS2, SS3-DS3, and SS4-DS4. The start of a SS phase (or the end of a DS phase) is always defined by the takeoff of the right foot, and the end of a SS phase (or the start of a DS phase) by the touchdown. In some references, such as Gutiérrez et al. (2002), the DS4 phase is called release phase, and in other works, such as Isele and Nixdorf (2010) and Isele et al. (2012), the start of the SS1 phase is defined as EoS (end of swings).

As reported in Tiedemann et al. (2022b) and Sánchez-Moreno et al. (2025), the variable that allows detection of the vertical movement of the feet is the Z-axis acceleration defined in the Earth reference frame. In this case, only the acceleration of the right foot must be analyzed because the left foot is always in contact with the ground.

The first step to obtain the correct Z-axis acceleration is to transform the right foot acceleration from the IMU reference frame to the Earth frame and subtract the gravity component. Thus,

$$a_{Earth}\left(t\right)=R_{IMU}^{Earth}\left(t\right)a_{IMU}\left(t\right)-{\left[0\;0\;9.81\right]}^{T},$$

with *t* = *h, 2h, …, t_release_*

where h is the sampling time, $a_{IMU}\left(t\right)={\left[a_{IMU_{x}}\left(t\right)\;a_{IMU_{y}}\left(t\right)\;a_{IMU_{z}}\left(t\right)\right]}^{T}$ denotes the acceleration vector expressed in the IMU reference frame, $a_{Earth}\left(t\right)={\left[a_{Earth_{x}}\left(t\right)\;a_{Earth_{y}}\left(t\right)\;a_{Earth_{z}}\left(t\right)\right]}^{T}$ denotes the acceleration vector expressed in the Earth reference frame, and $R_{IMU}^{Earth}\left(t\right)$ is the rotation matrix using the ENU convention. So,

$$R_{IMU}^{Earth}\left(t\right)=\left[\begin{array}{ccc} \cos\;\theta \left(t\right)\;\cos\;\psi \left(t\right) & \cos\;\psi \left(t\right)\;\sin\;\theta \left(t\right)\;\sin\;\phi \left(t\right)-\sin\;\psi \left(t\right)\;\cos\;\phi \left(t\right) & \cos\;\psi \left(t\right)\;\sin\;\theta \left(t\right)\;\cos\;\phi \left(t\right)+\sin\;\psi \left(t\right)\;\sin\;\phi \left(t\right)\\ \cos\;\theta \left(t\right)\;\sin\;\psi \left(t\right) & \sin\;\psi \left(t\right)\;\sin\;\theta \left(t\right)\;\sin\;\phi \left(t\right)+\cos\;\psi \left(t\right)\;\cos\;\phi \left(t\right) & \sin\;\psi \left(t\right)\;\sin\;\theta \left(t\right)\;\cos\;\phi \left(t\right)-\cos\;\psi \left(t\right)\;\sin\;\phi \left(t\right)\\ -\sin\;\theta \left(t\right) & \cos\;\theta \left(t\right)\;\sin\;\phi \left(t\right) & \cos\;\theta \left(t\right)\;\cos\;\phi \left(t\right) \end{array}\right]$$

where the Euler angles $\left(\psi ,\theta ,\phi \right)$ define the orientation by rotations around the Z-axis, Y-axis, and X-axis, respectively. Thus, the signal of interest to detect the right foot movement is:

$$a_{Earth_{z}}=\left[a_{Earth_{z}}\left(t_{start}\right),...,a_{Earth_{z}}\left(t_{release}\right)\right].$$

The takeoffs and touchdowns are defined by the local maxima of the $a_{Earth_{z}}$ signal within the temporal window delimited by *t*_*start*_ and *t*_*release.*_. To detect these maxima, it is important to observe that at each takeoff (R_*off*_), the hammer is moving upward from an LP to an HP, and at each touchdown (R_*down*_), the hammer is going downward from an HP to an LP. Thus, the detection of the maximum associated with a takeoff is performed by searching for the Z-axis acceleration peak between the two-time instants corresponding to a minimum (an LP) and its consecutive maximum (an HP) in the elevation angle of the wrist, that is, its Euler-Y orientation angle. A similar search is performed for finding the maxima associated with the touchdowns, but in this case looking for the peaks between the time instants defined by a maximum (an HP) and its consecutive minimum (an LP) in the Euler-Y angle of the wrist.

**FIGURE 5:**
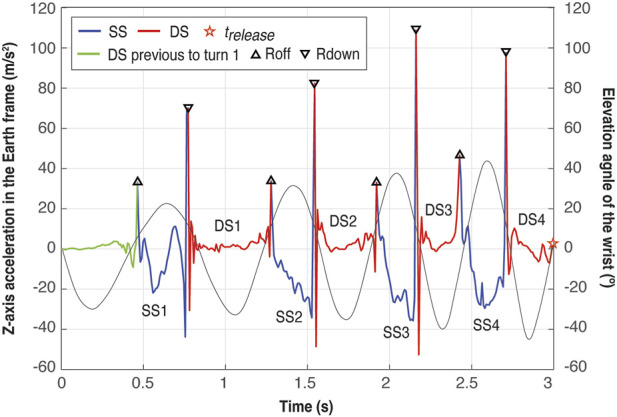
Example of phase delimitation using the right foot Z-axis acceleration maxima. The sinusoidal signal corresponds to the Euler-Y orientation angle of the wrist.

Figure 5 shows an example of the foot events detected within the temporal windows delimited by the minima and maxima of the wrist elevation angle. Once the time instants of R_*off*_ and R_*down*_ are known, the temporal characterization of the 4 SS and DS phases is defined as:

$$\text{Duration SS}i=t_{Rdown\_i}-t_{Roff\_i}$$

$$\text{ Duration DS}i=t_{Roff\_i+1}-t_{Rdown\_i}$$

with *i* = 1, 2, 3, and

$\text{Duration SS}4=t_{Rdown\_4}-t_{Roff\_4}\text{ Duration DS}4=t_{release}-t_{Rdown\_4}$

The throw shown in Figure 5 is the same of Figures 3 and 4.

### 4.3 Detection of LPs, HPs, and RP angles

Once the characterization of the SS and DS phases is complete, the detection of the four HPs, the four LPs, and the RP angles can be easily accomplished. The temporal instants corresponding to the maxima and minima of the wrist elevation angle have been previously identified, thus the HP and LP angles are given by the Euler-Y angle at these eight temporal instants: the maxima correspond to the HPs, and the minima to the LPs. The RP angle is obtained by the Euler-Y angle at *t*_*release*_.

**FIGURE 6:**
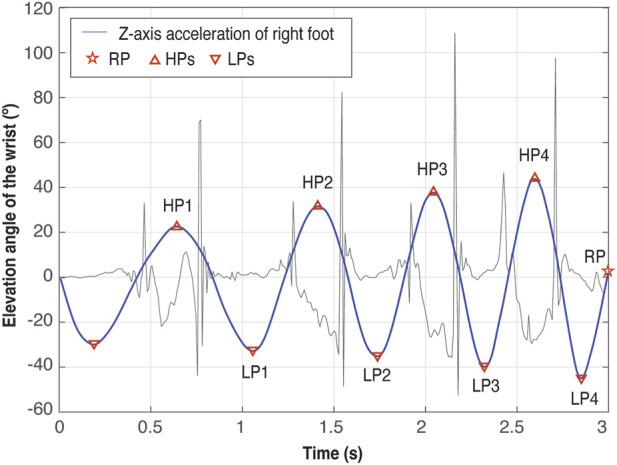
Example of detection of HPs, LPs, and RP angles.

To obtain the most accurate measurement possible, it is important to verify that the IMU is correctly positioned on the wrist, with its X-axis aligned along the arm’s longitudinal axis and securely attached over the distal radius bone to minimize soft tissue artifacts and measurement bias. Figure 6 shows the HPs, LPs, and RP angles of the previously analyzed throw.

### 4.4 Calculation of the tilt of the wrist orbits

**FIGURE 7:**
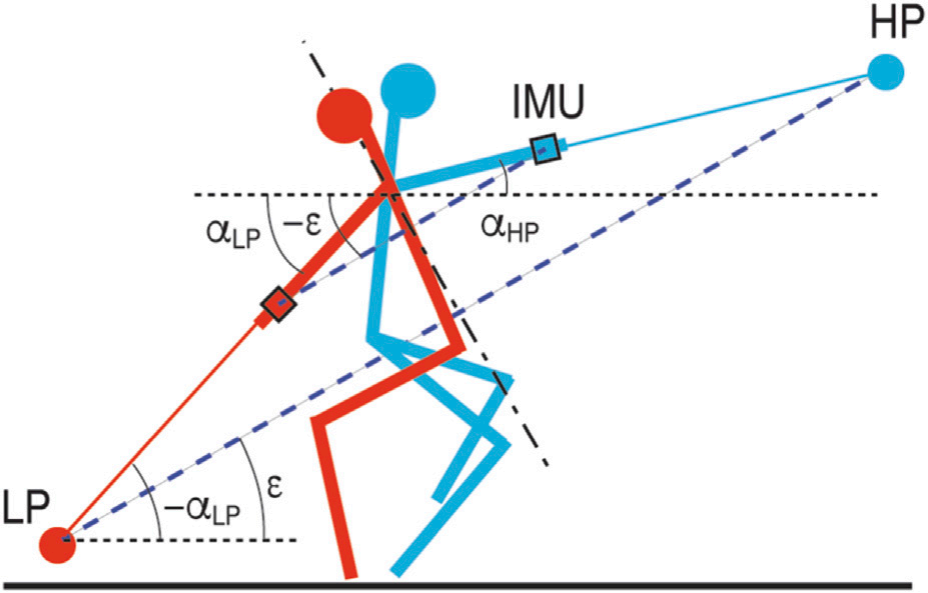
Simplification of the geometry of the hammer motion in a turn. Redrawn by the authors based on the geometric model described in Strelnitski (2020).

An interesting parameter to derive from the elevation angles is the inclination of the plane containing the orbit of the wrist. Geometrically, this parameter could be considered an approximation of the evolution of the tilt of the hammer orbits, that is, the inclination of the planes in which the path of the hammer head lies along the turns (Figure 7).

However, to assume that the elevation angles at the HP and LP time instants determine the tilt of the orbits is incorrect. According to Strelnitski (2020), it would be inaccurate to assume that the hammer wire lies in the plane of the hammer head’s orbit because this depends on the experience of the thrower. As shown in Figure 7, the projection of the ball’s orbit lies on a plane, while the wire and arms describe a conical surface above it.

Let us assume that in each turn ball and wrist move along parallel planes in circular orbits, both inclined with respect to the horizontal plane an angle ε . This assumption is a simplification because the thrower is moving across the circle, producing a non-circular orbit, and also because the rotation axis of the athlete–hammer system is located somewhere in the space between the athlete’s body and the hands. Figure 7 presents a simplification of the geometry of a turn with the rotation axis through the thrower. This orbit must be progressively steepening by the athlete in each turn to reach the optimal release angle of the hammer, which is considered as 44°–45° by most coaches. The tilt ε of each turn can be determined by its HP and LP angles. Thus,

$$\varepsilon_{wrist\_i}=\frac{\alpha_{HP\_i}-\alpha_{LP\_i}}{2}$$

with i=1,2,3,4 . Using this definition, the example throw presented above showed a gradual increase in the wrist orbit tilt: 27°, 33°, 38°, and 44°. This progression corresponds to good technique, as the thrower progressively steepens the orbit throughout the turns to reach an optimal release angle of the hammer.

### 4.5 Detection of the pelvic inclination

One additional biomechanical parameter that can be obtained with the IMU located at the lumbar zone is the pelvic inclination using the Euler-Y orientation. Similarly to the wrist IMU, it is necessary to perform a calibration procedure at the beginning of each throw to compensate for the initial offset in the Euler-Y angle of the IMU due to the athlete’s movements. This procedure is executed at the same time as the wrist IMU calibration, and the reference posture is used to establish the zero-degree inclination baseline:

$$pelvic\_inclination\_offset=\frac{1}{100}\sum_{i=1}^{100}Euler\_Y\_pelvic\left(i*h\right)$$

$$Euler\_Y\_pelvic\left(t\right)=Euler\_Y\_pelvic\left(t\right)-pelvic\_inclination\_offset$$

with $t=h,2h,...,t_{release}$ .

**FIGURE 8:**
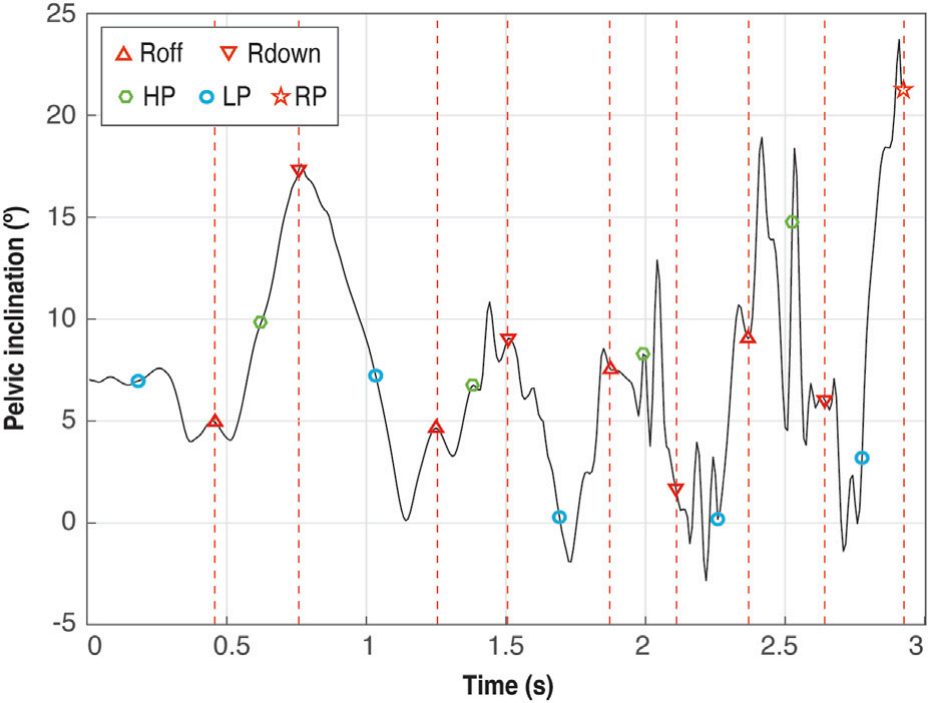
Pelvic inclination during a throw. Positive values indicate backward tilt.

Figure 8 shows an example of the pelvic inclination evolution during a trial where an initial offset of 3.4° was applied. It should be noted that at *t* = 0 the inclination is not zero because the visualization of the throw starts when the athlete is already moving the hammer (see Section 4.1), finishing the second swing before starting the first SS phase. In the figure, a backward tilt of the thrower can be appreciated at the end of the last turn to achieve a high hammer elevation angle, ready for release.

### 4.6 Detection of azimuthal angles

One of the most relevant parameters in the biomechanical analysis of any hammer throw is the azimuthal angle. Specifically, these angles describe the position of the hammer head within the throwing circle at critical events (see Figure 1), i.e., takeoff, touchdowns, HPs, LPs, and RP. Although the IMU placed on the wrist can measure azimuthal angles through Euler-Z angle calculations, some limitations must be acknowledged. First, the sensor measures the wrist orientation rather than the hammer head position directly, assuming both are aligned during the rotations, an assumption that may not hold perfectly due to cable elasticity, particularly during high-acceleration phases and at release. Second, gyroscope drift accumulation during multiple rotations can introduce progressive errors in azimuthal angle estimation, which the initial calibration procedure cannot fully eliminate during dynamic movement.

However, despite these constraints, the wrist-mounted IMU offers significant advantages for practical hammer throw analysis. The system provides continuous, real-time measurement of angular positions throughout the entire throwing sequence without the spatial constraints or occlusion issues inherent in optical motion capture systems. This enables field-based assessments during actual training sessions, allowing coaches and athletes to obtain immediate feedback on critical parameters such as symmetry, consistency across rotations, and relative angular positions at key events.

In our setup, these angles are obtained from the Euler-Z orientation angle of the wrist IMU at the temporal instants corresponding to the critical events. It is necessary to apply a calibration procedure before each throw to compensate for the initial offset in the Euler-Z angle of the wrist IMU relative to the 0° position of the circle. At the start of each recording, the thrower remained standing still in an upright position with the arm held horizontally (see Figure 2) and aligned with the 0°–180° axis of the circle. The orientation offset is obtained by averaging the first 100 samples of the recording (it is equivalent to 0.833 s). After a brief period, the athlete initiated the throwing movements. Thus, the orientation offset is calculated as follows:

$$orientation\_offset=\frac{1}{100}\sum_{i=1}^{100}Euler\_Z\_wrist\left(i*h\right)$$

Then, the compensations were applied as:

$$Euler\_Z\_wrist\left(t\right)=Euler\_Z\_wrist\left(t\right)-orientation\_offset$$

with $t=h,2h,...,t_{release}$ .

The azimuthal angle at any critical event occurring at time $t_{event}$ is defined as:

$$\varphi \left(t_{event}\right)=Euler\_Z\_wrist\left(t_{event}\right)$$

where $t_{event}$ corresponds to the time instant of any critical event (R_*off*_, HP, R_*down*_, LP, or RP) identified in Sections 4.2 and 4.3. The resulting angle φ represents the angular position of the wrist projected onto the horizontal plane of the throwing circle, measured from the 0° reference axis defined during the initial calibration posture (see Figure 1).

**FIGURE 9:**
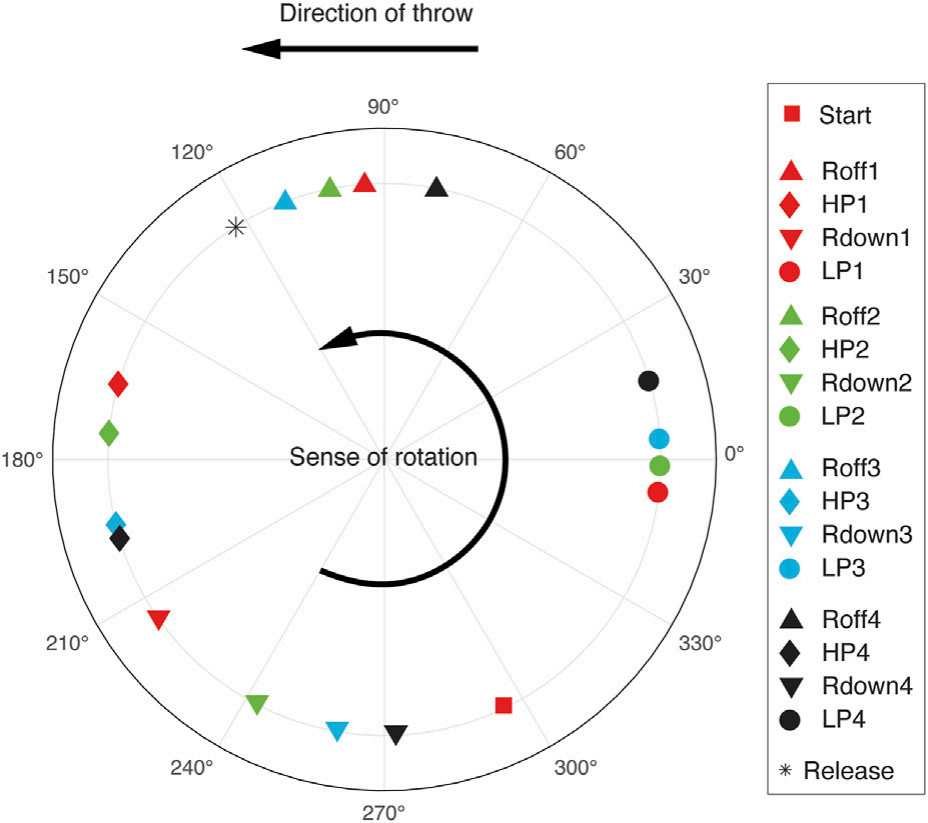
Azimuthal angles of the wrist at the critical events.

Figure 9 shows the azimuthal angles corresponding to the throw used as an example in previous figures. The initial offset of the Euler-Z angle to align the wrist IMU orientation with the 0° angle of the circle was 27°.

### 4.7 Calculation of angular speed and linear acceleration magnitude

A biomechanical parameter that can be easily obtained from the data of the IMU located at the wrist is the linear acceleration magnitude. The steps to transform the wrist acceleration from the IMU reference frame to the Earth frame are the same as for the right foot acceleration, that is,

$$a_{Earth}\left(t\right)=R_{IMU}^{Earth}\left(t\right)a_{IMU}\left(t\right)-{\left[0\;0\;9.81\right]}^{T},\text{with }t=h,2h,\ldots t_{release}$$

Then, the linear acceleration magnitude at each time instant is calculated as:

$$a_{magnitude}\left(t\right)=\sqrt{a_{Earth_{x}}^{2}\left(t\right)+a_{Earth_{y}}^{2}\left(t\right)+a_{Earth_{z}}^{2}\left(t\right)}$$

The angular velocity magnitude of the wrist is obtained directly from the IMU data without further processing:

$$\omega_{magnitude}\left(t\right)=\sqrt{\omega_{{IMU}_{x}}^{2}\left(t\right)+\omega_{{IMU}_{y}}^{2}\left(t\right)+\omega_{{IMU}_{z}}^{2}\left(t\right)}$$

**FIGURE 10:**
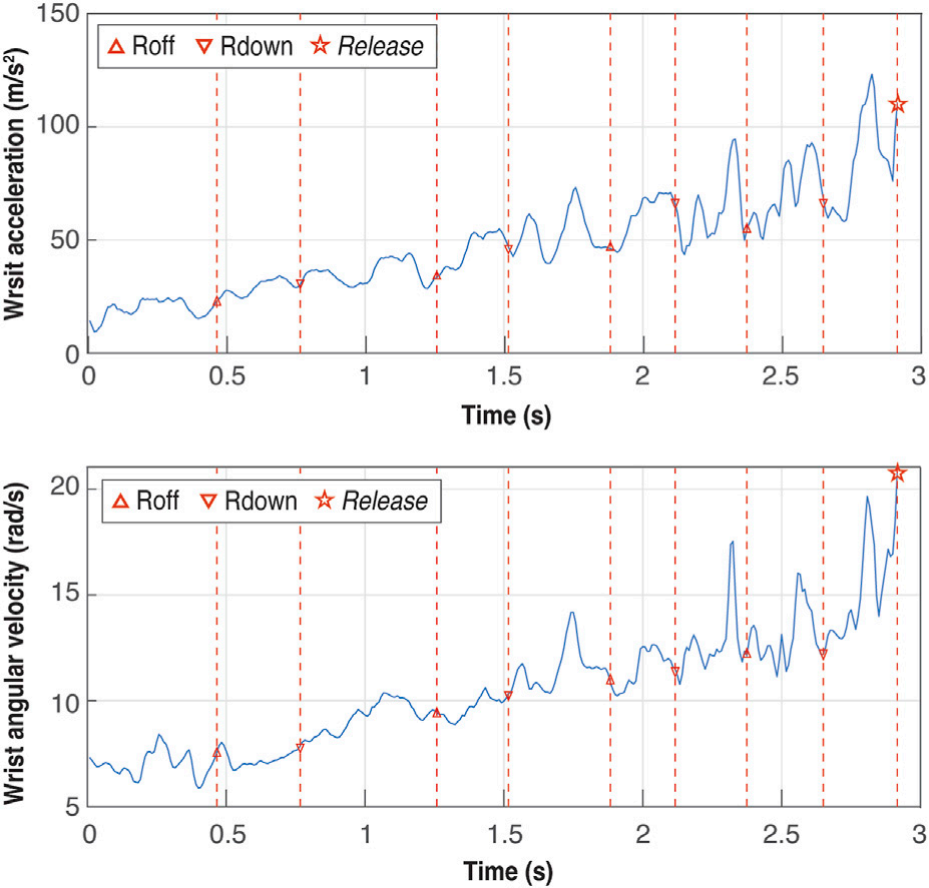
Linear acceleration magnitude and the angular velocity of the wrist during a throw.

Because angular velocity is a free vector, it represents the same physical rotation regardless of the reference frame used to express it. Thus, it is not necessary to transform the angular velocity from the IMU body frame to the Earth frame. Figure 10 shows these two parameters, displaying the characteristic pattern of progressive increase in both linear acceleration magnitude and angular velocity during the movement.

Small differences exist between wrist and hammer head kinematics since the hammer + thrower system is not a true rigid body. The hammer wire (970 mm–992 mm) introduces flexibility into the system, allowing for relative motion between the thrower’s hands and the hammer head. Additionally, the thrower’s body segments (arms, trunk, and legs) exhibit independent movements that are not perfectly transmitted to the hammer. Despite these sources of kinematic variability, the wrist remains mechanically coupled to the hammer through the wire and handle, meaning that the general acceleration trends and phase characteristics observed at the wrist closely mirror those of the hammer head. Therefore, wrist-mounted IMU data can serve as a practical proxy for understanding the hammer’s acceleration behavior, particularly when direct measurement of the hammer head is not feasible or would interfere with the throwing technique.

## 5 Validation phase

The biomechanical parameters obtained from the IMU system, including support phase durations, angular speed, linear acceleration magnitude, pelvic inclination, and azimuthal angles, do not require external validation against optical motion capture systems, provided that the initial calibration procedure to eliminate sensor offsets is correctly performed. Modern MEMS-based IMUs, such as the Movella DOT used in this study, are factory-calibrated devices that directly measure physical quantities with high accuracy within their specified operating ranges. Recent validation studies have demonstrated that Movella DOT sensors achieve excellent concurrent validity when compared to gold-standard optoelectronic systems. Cudejko et al. (2022) reported fair to excellent validity of orientations and accelerations measured with the Xsens DOT during functional activities including squats, jumps, walks, and stair ambulation. Beange et al. (2024) validated the Xsens DOT for tracking multiplanar spine movement. More recently, Villa et al. (2025) demonstrated that a multi-sensor Movella DOT system achieved strong correlations, low RMSE values, and negligible systematic biases compared to optical motion capture across a variety of full-body tasks.

The onboard Kalman-based sensor fusion of the Xsens DOT computes orientation estimates that, when properly referenced to a known initial angular position, provide reliable measurements without drift accumulation over the short duration of a hammer throw (typically less than 3 s). Therefore, the validity of these parameters depends on the correct execution of the offset calibration at the beginning of each trial rather than on comparison with external reference systems. However, despite these claims that IMU data do not require external validation, a validation phase was undertaken to ensure the reliability of the results obtained in this work.

### 5.1 Validation using a motion capture system

Tables 2–5 present the results obtained with the VICON and IMU systems corresponding to the temporal and angular amplitude of the phases and the elevation angle of the left arm. It should be noted that in these indoor experiments the hammer could not be released. Therefore, it was considered that the end of DS4 was defined as the instant when the right foot left the ground during the thrower’s deceleration turns.

**TABLE 2:** Duration (in seconds) of each SS and DS phase across the four turns.

Phase	Throw 1		Throw 2		Throw 3	
​	IMU	MOCAP	IMU	MOCAP	IMU	MOCAP
SS1	0.31	0.26	0.30	0.32	0.24	0.26
DS1	0.60	0.65	0.52	0.48	0.59	0.58
SS2	0.22	0.19	0.26	0.23	0.23	0.20
DS2	0.45	0.46	0.37	0.38	0.42	0.45
SS3	0.23	0.22	0.25	0.25	0.23	0.21
DS3	0.31	0.34	0.30	0.31	0.28	0.28
SS4	0.24	0.22	0.22	0.22	0.23	0.22
DS4	0.29	0.32	0.26	0.29	0.23	0.26
Total time	2.65	2.66	2.48	2.48	2.45	2.47

**TABLE 3:** Angular displacement (in degrees) of each SS and DS phase across the four turns.

Phase	Throw 1		Throw 2		Throw 3	
​	IMU	MOCAP	IMU	MOCAP	IMU	MOCAP
SS1	102°	83°	105°	113°	86°	87°
DS1	260°	279°	245°	232°	273°	273°
SS2	98°	85°	128°	108°	110°	95°
DS2	242°	251°	222°	230°	245°	264°
SS3	129°	122°	152°	150°	135°	121°
DS3	205°	220°	211°	221°	200°	194°
SS4	156°	139°	150°	140°	157°	154°
DS4	214°	233°	198°	219°	179°	194°
Total angular path	1,406°	1,412°	1,411°	1,413°	1,385°	1,385°

**TABLE 4:** Angles (in degrees) corresponding to the HPs and LPs across the four turns.

Turn	​	Throw 1		Throw 2		Throw 3	
​	Critical instant	IMU	MOCAP	IMU	MOCAP	IMU	MOCAP
1	HP	7°	8°	8°	7°	11°	10°
	LP	−25°	−27°	−21°	−25°	−24°	−28°
2	HP	20°	19°	19°	18°	19°	18°
	LP	−27°	−29°	−24°	−28°	−25°	−29°
3	HP	26°	26°	28°	27°	26°	24°
	LP	−30°	−33°	−31°	−36°	−28°	−32°
4	HP	35°	34°	36°	34°	34°	31°
	LP	−33°	−40°	−33°	−40°	−31°	−37°

**TABLE 5:** Agreement statistics between IMU and MOCAP systems.

Variable	N	ICC	95% CI	r	Bias	95% LoA	RMSE	MAE	Cohen’s d
Phase duration (s)	24	0.977	[0.947,0.990]	0.978	−0.001	[−0.053, 0.051]	0.026	0.022	−0.03
Angular displacement (°)	24	0.976	[0.946,0.990]	0.985	−0.2	[−27.0, 26.6]	13.4	11.8	−0.02
HP elevation (°)	12	0.989	[0.964,0.997]	0.997	1.1	[−0.9, 3.0]	1.4	1.2	1.09
LP elevation (°)	12	0.649	[0.327,0.868]	0.969	4.3	[1.1, 7.6]	4.6	4.3	2.60
HP + LP combined (°)	24	0.992	[0.982,0.997]	0.999	2.7	[-1.5, 6.9]	3.4	2.8	1.27

ICC: intraclass correlation coefficient; LoA: limits of agreement; RMSE: root mean square error; MAE: mean absolute error; HP: high point; LP: low point.

Agreement between the IMU-based and VICON systems was assessed using Bland-Altman analysis and intraclass correlation coefficients (ICC). The ICC(2,1) model (two-way random effects, absolute agreement, single measures) was used to evaluate the reliability between measurement systems. ICC values were interpreted according to established guidelines: values below 0.50 indicate poor reliability, 0.50–0.75 moderate reliability, 0.75–0.90 good reliability, and above 0.90 excellent reliability.

#### 5.1.1 Phase duration

The comparison of phase durations between IMU and MOCAP systems (Table 2) revealed excellent agreement, with an ICC of 0.977 (95% CI: 0.947–0.990) and a Pearson correlation coefficient of r = 0.978 (p < 0.001). Cohen’s d was −0.03, indicating negligible systematic difference between systems. Bland-Altman analysis showed a negligible mean bias of −0.001 s with 95% limits of agreement (LoA) ranging from −0.053 to 0.051 s. The root mean square error (RMSE) was 0.026 s and the mean absolute error (MAE) was 0.022 s, indicating that temporal measurements from the IMU system are highly consistent with the reference MOCAP system.

#### 5.1.2 Angular displacement

The angular displacement of each phase (Table 3) also demonstrated excellent agreement between systems, with an ICC of 0.976 (95% CI: 0.946–0.990) and r = 0.985 (p < 0.001). Cohen’s d was −0.02, confirming negligible systematic bias. The Bland-Altman analysis revealed a mean bias of −0.2° with 95% LoA from −27.0° to 26.6°. The RMSE was 13.4° and the MAE was 11.8°. While the limits of agreement appear relatively wide in absolute terms, they represent approximately 8% of the typical phase angular displacement (ranging from 85° to 280°), which is acceptable for this application.

#### 5.1.3 Arm elevation angles

The analysis of high points (HP) and low points (LP) of arm elevation (Table 4) showed different levels of agreement. For HP angles, the agreement was excellent (ICC = 0.989, 95% CI: 0.964–0.997; r = 0.997, p < 0.001; Cohen’s d = 1.09), with a mean bias of 1.1° and narrow 95% LoA from −0.9° to 3.0°. The RMSE and MAE were 1.4° and 1.2°, respectively. In contrast, LP angles showed moderate agreement (ICC = 0.649, 95% CI: 0.327–0.868; r = 0.969, p < 0.001; Cohen’s d = 2.60), with a systematic bias of 4.3° indicating that the IMU consistently measured less negative values than MOCAP. The large effect size confirms a meaningful systematic difference at the low points. The 95% LoA ranged from 1.1° to 7.6°, with RMSE = 4.6° and MAE = 4.3°. When considering HP and LP combined, the overall agreement was excellent (ICC = 0.992, 95% CI: 0.982–0.997; r = 0.999, p < 0.001; Cohen’s d = 1.27), with a mean bias of 2.7° and 95% LoA from −1.5° to 6.9°. It should be noted that the Pearson correlation coefficient r, while reported for completeness and comparability with previous studies, measures linear association rather than agreement and may overestimate consistency in the presence of systematic bias, as illustrated by the LP data (r = 0.969 vs. ICC = 0.649). From a practical standpoint, since the LP bias was consistent across all trials (mean: 4.3°, 95% LoA: 1.1°–7.6°), the LP angles measured by the wrist IMU can be interpreted as conservative estimates. Coaches should expect the actual hammer head LP angles to be approximately 4°–5° more negative than the wrist-measured values.

Additionally, qualitative comparison of the angular velocity and linear acceleration waveforms revealed consistent temporal patterns between the IMU and MOCAP systems. Both signals exhibited similar peak magnitudes and timing across the four turns, supporting the validity of the IMU-derived kinematic data for hammer throw analysis. For the sake of brevity, figures and detailed comparisons of these continuous signals are not included in this paper.

### 5.2 Validation of the elevation angle of the wrist using an additional IMU

The elevation angles of the wrist IMU require a specific analysis because the wrist and the hammer head are connected through a flexible wire (970–992 mm), and their elevation angles may differ depending on the phase of the throw and the wire tension. Also, for the same reason, it is necessary to compare the patterns of the angular speed of the hammer and the wrist.

To observe whether this discrepancy exists, a specific validation session was conducted in which an additional IMU was installed on the hammer to compare the elevation angles measured at both locations. The additional IMU was placed at the end of the wire close to the ball, with the X-axis pointing toward the thrower. Athlete ID 1 was selected due to his higher proficiency and experience, and five trials were performed during the validation session.

**FIGURE 11:**
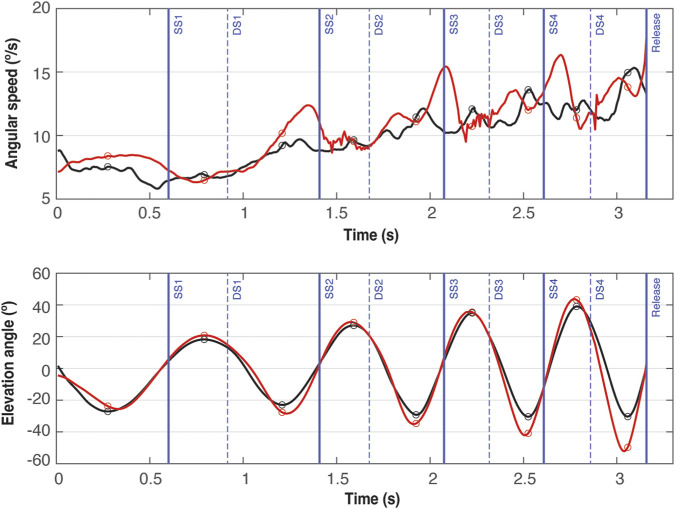
Angular velocity and elevation angle of the wrist (black) and hammer wire (red). Circles indicate the time instants of the HPs and LPs of the wrist.

Figure 11 shows the angular velocity and elevation angles of both the hammer (red) and the wrist-mounted IMU (black) throughout the best throw of the validation session with 53.25 m (the other throws showed similar results in the patterns of angular speeds and angles). The key observation from Figure 11 is that discrepancies in elevation angles occur mainly during the DS phases, and during the SS phases the elevation of the wrist can be used as a proxy for the elevation angle of the hammer head with only minor discrepancies.

**FIGURE 12:**
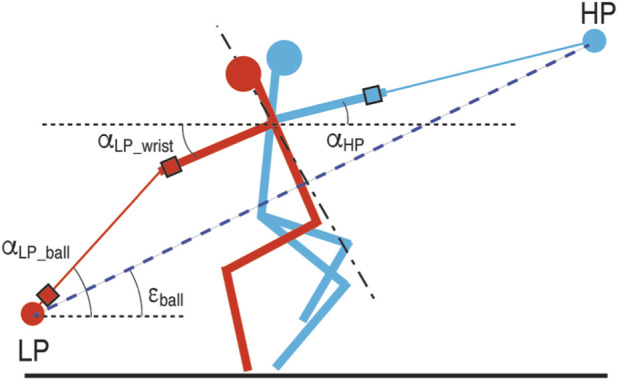
Simplification of the geometry of the hammer motion in a turn showing the differences between the elevation angle of wire and wrist at the LP. Redrawn by the authors, extending the geometric model described in Strelnitski (2020) to include the elevation angle differences between wire and wrist at the LP.

After examining the setups and the lateral video recordings in each throw (a GoPro HERO 13 camera at 120 fps was used), it was detected that the difference at the DS phases was real and not a problem of the IMU calibrations or installations. Figure 12 shows a modification of Figure 7 that includes the elevation angle of the hammer in the geometry of the turn.

Analyzing Figure 12, it is possible to estimate the tilt of the hammer orbit in each turn, that is, $\varepsilon_{ball\_i}$ . As said before, considering that the $\alpha_{HP}$ of the wrist and hammer are equal, we have:

$$\varepsilon_{ball\_i}=\arccos\left(\frac{A}{\sqrt{A^{2}+B^{2}}}\right)$$

or:

$$\varepsilon_{ball\_i}=\arctan\left(\frac{B}{A}\right)$$

with:

$$A=h_{arm}\cos\left(\alpha_{LP\_wrist\_i}\right)+h_{hammer}\cos\left(\alpha_{LP\_ball\_i}\right)+\left(h_{arm}+h_{hammer}\right)\cos\left(\alpha_{HP\_i}\right)B=h_{arm}\sin\left(\alpha_{LP\_wrist\_i}\right)+h_{hammer}\sin\left(\alpha_{LP\_ball\_i}\right)+\left(h_{arm}+h_{hammer}\right)\sin\left(\alpha_{HP\_i}\right)$$

where $h_{arm}$ is the length of the arm from the shoulder to the knuckles where the athlete grips the handle, and $h_{hammer}$ is the length of the hammer, from the handle to the center of the ball. It must be noted that if $\alpha_{LP\_wrist\_i}=\alpha_{LP\_ball\_i}$ , the expression of $\varepsilon_{ball\_i}$ reduces to the expression of the geometry of Figure 7. Using the angles from Figure 11 and the data of Athlete ID 1 ( $h_{arm}$ = 0.76 m, $h_{hammer}$ = 1.155 m), it is obtained that the tilt of the hammer orbit in the four turns was 22°, 30°, 36°, and 41°. This formula is intended for practical computation when a hammer-mounted IMU is available, providing the $\alpha_{LP\_ball\_i}$ values required for the calculation.

Despite the discrepancies observed in elevation angles, the wrist-mounted IMU provides valuable information for hammer throw analysis, particularly during the SS phases. In these phases, the wrist IMU serves as a reliable proxy for hammer kinematics, capturing elevation angles with good accuracy. During the DS phases, however, the wrist systematically underestimates the hammer elevation amplitude at the LPs.

The discrepancies observed in angular speed between the wrist and hammer head in Figure 11 are normal and can be explained by the relationship between angular velocity, radius of rotation, and linear velocity. During the DS phases, the thrower actively applies torque to the hammer through trunk torsion relative to the hips, creating a “wound-up position” where the body leads the hammer, facilitating greater hammer acceleration (Dapena and McDonald, 1989). This angular lag between the wrist and hammer, combined with the flexible wire connection, allows the hammer to experience higher angular velocities than the wrist during these active phases. In the later turns (SS3-SS4), the discrepancy persists even during passive phases because the accumulated angular momentum of the hammer is sufficiently large that it no longer follows the wrist motion exactly (Castaldi et al., 2022).

## 6 Biomechanical parameters obtained from the athletes

To verify that the biomechanical parameters obtained with the IMU-based procedure were within expected ranges, Tables 6–9 present temporal and angular variables that can be compared against sex-specific reference values from the scientific literature. For the sake of brevity, only the data of the longest throw of each of the five athletes are presented in the tables.

**TABLE 6:** Duration (in seconds) of the SS and DS phases across the four turns.

Phase	Athlete 1	Athlete 2	Athlete 3	Athlete 4	Athlete 5
SS1	0.30	0.29	0.26	0.31	0.33
DS1	0.47	0.50	0.44	0.34	0.45
SS2	0.26	0.22	0.21	0.25	0.27
DS2	0.31	0.38	0.31	0.28	0.37
SS3	0.29	0.24	0.19	0.27	0.27
DS3	0.26	0.28	0.29	0.25	0.33
SS4	0.26	0.27	0.21	0.25	0.28
DS4	0.28	0.25	0.35	0.25	0.24
Total time	2.42	2.42	2.26	2.20	2.52

**TABLE 7:** Azimuth angles at critical instants across the four turns.

Turn	Critical instant	Athlete 1	Athlete 2	Athlete 3	Athlete 4	Athlete 5
1	Takeoff	70°	76°	81°	112°	124°
	HP	150°	149°	146°	159°	161°
	Touchdown	192°	201°	212°	257°	257°
	LP	341°	343°	335°	354°	350°
2	Takeoff	71°	95°	101°	98°	115°
	HP	162°	172°	179°	179°	161°
	Touchdown	216°	216°	234°	244°	253°
	LP	352°	21°	3°	1°	7°
3	Takeoff	52°	91°	80°	78°	102°
	HP	174°	194°	183°	180°	177°
	Touchdown	237°	246°	209°	256°	257°
	LP	0°	21°	14°	4°	0°
4	Takeoff	57°	89°	62°	84°	105°
	HP	184°	206°	198°	187°	181°
	Touchdown	238°	267°	205°	261°	268°
	LP	15°	16°	16°	354°	0°
Delivery	​	90°	95°	113°	106°	105°

**TABLE 8:** Angles (in degrees) corresponding to the HPs, LPs, and RP across the four turns.

Turn	Critical instant	Athlete 1	Athlete 2	Athlete 3	Athlete 4	Athlete 5
1	HP	17°	27°	20°	9°	26°
	LP	−30°	−29°	−31°	−32°	−26°
2	HP	26°	30°	27°	15°	30°
	LP	−36°	−32°	−36°	−34°	−26°
3	HP	35°	32°	28°	21°	32°
	LP	−40°	−38°	−37°	−42°	−33°
4	HP	40°	31°	29°	28°	39°
	LP	−38°	−35°	−40°	−42°	−39°
Release	RP	−2°	−8°	−1°	2°	5°

**TABLE 9:** Tilt (in degrees) of the wrist orbit across the four turns.

Turn	Athlete 1	Athlete 2	Athlete 3	Athlete 4	Athlete 5
1	23°	28°	26°	21°	26°
2	31°	31°	31°	25°	28°
3	37°	35°	32°	31°	32°
4	39°	33°	34°	36°	39°

The biomechanical parameters obtained are consistent with findings from the scientific literature (Bartonietz, 2008; Brice et al., 2018b; Dinsdale et al., 2017a; Dinsdale et al., 2017b; Gutiérrez et al., 2002; Huang et al., 2024; Isele and Nixdorf, 2010; Isele et al., 2012; Judge et al., 2016; Liu et al., 2025; Panoutsakopoulos et al., 2012; Pavlović, 2020), further supporting the validity of the proposed IMU-based methodology for field-based biomechanical assessment. A detailed biomechanical analysis is beyond the scope of this paper. Nevertheless, the parameters obtained with the proposed methodology are directly related to throwing performance according to the literature. For instance, the progressive increase of the orbit tilt across turns determines the release angle, which is considered optimal at 44°–45° (Strelnitski, 2020). Additionally, larger azimuthal displacements during the DS phases have been associated with higher hammer velocity at release (Rojas-Ruiz and Gutiérrez-Dávila, 2009).

## 7 Discussion

While extensive 3D motion capture technologies exist, coaches often find them too cumbersome to be useful for regular training. The purpose of this study was to present a low-cost, time-efficient diagnostic solution for hammer throw that avoids the limitations of conventional methods based on video analysis, namely, the limited accessibility and the significant investment in time and money.

This study has limitations. Although the validation against the VICON system was limited to three throws from a single athlete, which constitutes a limitation of this study, several factors support the reliability of the results: the selected athlete was the most experienced in the study (official PB: 63.95 m), ensuring technically consistent throws; the three trials yielded 24 phase duration and 24 angular displacement measurements for statistical analysis; the field testing involved five national-level athletes performing six throws each, demonstrating the system’s applicability across different throwers; and the biomechanical parameters obtained were consistent with sex-specific reference values from the scientific literature (Tables 6–9). Future validation sessions with additional athletes are planned to further strengthen these results. The three-IMU setup cannot provide all the kinematic parameters that a motion capture system can (e.g., twisting and trailing angles, 3D path of the hammer), and some results may present significant errors (e.g., 3D path of the trunk or arm due to double integration of accelerations). Additionally, the analysis revealed discrepancies between wrist and hammer head elevation angles, particularly during the DS phases. Nevertheless, even in these phases, the wrist signal preserves the fundamental oscillation pattern. When reviewing the literature for a biomechanical explanation of the differences between both angles, it was found that the elevation of the wrist has not been measured and compared with that of the hammer until now. While the formula presented in Section 5.2 enables the practical computation of the hammer orbit tilt when a hammer-mounted IMU is used, the biomechanical explanation of why the elevation angles of the wrist and the hammer head differ, particularly during the DS phases, remains an open question to be addressed in further work. However, the results demonstrate that the wrist-mounted IMU provides reliable elevation data, making it a useful proxy for assessing the athlete’s technique, and that the incorporation of data from a hammer-mounted IMU enables the acquisition of reliable information on the tilt of the hammer orbit.

The validation against the VICON system confirmed that the IMU-based measurements are sufficiently accurate for practical coaching applications. The excellent agreement observed for phase durations and angular displacements indicates that the IMU system can reliably characterize the temporal and spatial structure of the throwing technique. Regarding arm elevation, the high points showed excellent agreement, while the low points exhibited a systematic bias. This discrepancy may be attributed to soft tissue artifacts at the wrist during high-acceleration phases when the arm reaches its lowest position, or to differences in the anatomical reference points between systems. Despite this limitation, the overall pattern of arm elevation is preserved, which is the most relevant information for coaching purposes.

It was not possible to compare our results with data from other IMU-based works on hammer biomechanics. The study by Tiedemann et al. (2022b), which used a 17-IMU full-body sensor suit with athletes of similar performance level to ours, does not report the values of kinematic parameters. All available references reporting relevant data, such as phase durations or azimuthal angles, are based on 3D video systems with world-level competitors as participants. However, the comparison between the biomechanical parameters obtained with the proposed IMU-based methodology and sex-specific reference values from the scientific literature confirmed that the system provides measurements within expected technical ranges, supporting the validity of the proposed approach for field-based biomechanical assessment of hammer throwing technique.

Despite these limitations, a set of relevant parameters for coaches can be obtained with high accuracy, providing fast feedback or serving as the basis for a more exhaustive biomechanical analysis.

## 8 Conclusions and further works

This study showed that a three-IMU setup can capture many of the most relevant literature-based biomechanical parameters of the hammer throwing technique without the need to access biomechanics laboratories or high-performance sports centers. The results obtained from the trials demonstrated the effectiveness of the three-IMU approach as an additional resource for analyzing athlete performance during regular training sessions or at specific moments in the annual training plan. The low-cost and portable nature of IMU technology makes biomechanical analysis accessible to a broader range of athletes and training environments, facilitating longitudinal monitoring of technical development over time.

Future work will focus on exploring the possibility of obtaining the twisting and trailing angles by combining information from the lumbar and wrist IMUs with a fourth IMU placed on the chest. The twisting angle is calculated as the difference in orientation angle between the hip axis and the shoulder axis, and the trailing angle is defined as the angle between the shoulder axis and the line connecting the hammer head to the shoulder axis. Additionally, a biomechanical study to explain the differences between the elevation angles of hammer and wrist will be addressed. To this end, further experiments with a hammer-mounted IMU are planned with a larger number of athletes to better analyze the hammer head kinematics and the relationship between its orbit tilt and angular velocity. Furthermore, an extended data collection campaign is underway to build a comprehensive dataset that will enable a statistical analysis of the relationship between the IMU-derived parameters and the throwing distance.

## Data Availability

The raw data supporting the conclusions of this article will be made available by the authors, without undue reservation.

## Footnotes

1
https://movella.com

2
https://www.noitom.com/perception-neuron-series

3
https://www.synertial.com

4 Bluetooth Low Energy.

5
https://www.vicon.com
